# The Ellis–Baldwin test

**DOI:** 10.1098/rsta.2024.0027

**Published:** 2025-02-13

**Authors:** Nathan J. Secrest

**Affiliations:** ^1^ U.S. Naval Observatory, 3450 Massachusetts Ave. NW, Washington, DC 20392-5420, USA

**Keywords:** cosmological principle, large-scale structure, cosmic microwave background

## Abstract

The standard cosmological model ΛCDM is described by the Friedman–Lemaitre–Robertson–Walker (FLRW) metric, which requires that the universe be isotropic and homogeneous on large scales, an assumption called the Cosmological Principle. If this assumption is accurate, then the dipole anisotropy observed in the cosmic microwave background (CMB) corresponds to our motion with respect to large-scale structure at approximately 370 km s^−1^, which can be tested by measuring the corresponding dipole predicted in counts of cosmologically distant sources. This consistency test, first proposed in 1984 by Ellis & Baldwin, became possible in the twenty-first century with the advent of large catalogues of radio sources and quasars. Subsequent Ellis–Baldwin tests have consistently shown an anomalously large dipole, two to three times larger than predicted by the kinematic interpretation of the CMB dipole, which has recently reached a statistical significance of over 5σ. In these proceedings, I review the Ellis–Baldwin test, the key results that revealed this anomaly, and comment on the status of research on this problem, which threatens a foundational assumption underpinning FLRW-based cosmologies such as ΛCDM.

This article is part of the discussion meeting issue ‘Challenging the standard cosmological model’.

## 1. Background

The standard, Lambda cold dark matter (ΛCDM) cosmological model is built on the foundational assumption that, when averaged on large enough scales, the distribution of matter and energy in the universe is both isotropic and homogeneous. This assumption, called the Cosmological Principle, leads to to the Friedmann equations, which provide an exact solution to the Einstein field equations based on the Friedmann–Lemaitre–Robertson–Walker (FLRW) metric


(1.1) $$ds^{2}=dt^{2}-a^{2}(t)\left[\frac{dr^{2}}{1-kr^{2}}+r^{2}(d\theta^{2}+\sin^{2}\theta d\phi^{2})\right].$$

The evolution of the universe is thus described by a dimensionless, time-dependent scale factor a(t). This time parameter t is the time a clock with zero peculiar motion, moving with the expansion of the universe (the Hubble flow), would measure in the absence of time dilation by local variations in the density of matter and energy; i.e. it is the time in comoving coordinates. The assumption of isotropy and homogeneity ensures that t is a single value everywhere, giving the universe a definable age. The standard (or ‘concordance’) ΛCDM model is an instance of a Friedmann cosmology based on the FLRW metric so relies explicitly on assuming the Cosmological Principle.

Without this assumption, the fundamental parameters describing the universe, such as the baryonic and dark matter densities, the Hubble constant 100h km s^−1^ Mpc^−1^ and the age of the universe do not take on unique values that describe the universe as a whole. It is self-evidently true that isotropy and homogeneity must be *statistical* properties of the universe, as no observers could arise otherwise. Thus, the Friedmann equations describe the expansion or contraction of the universe only on ‘cosmological scales’, scales at which the peculiar velocities of clumped matter such as galaxies should be negligible compared with the Hubble flow. The commonly adopted cosmological scale in ΛCDM is approximately 100 h^−1^ Mpc.

While there is increasing evidence for anomalously large ‘bulk flows’ on scales considerably in excess of this (e.g. [1]), consistency tests of ΛCDM should nonetheless be feasible using still more distant matter. Indeed, with the tightest constraints on the parameters of ΛCDM set by the z∼1100 cosmic microwave background (CMB), any deviations from ΛCDM are by construction more likely to manifest at much later redshifts when the universe has had time to evolve. Evidence for an evolutionary history different from that predicted given the best-fit parameters of ΛCDM would motivate more serious consideration of a modified cosmological standard model or even new physics.

The test proposed four decades ago by Ellis & Baldwin [2] is a powerful, model-independent and elegantly simple method of testing these foundational assumptions. The CMB exhibits a prominent dipole anisotropy of amplitude $\Delta T/T∼10^{-3}$, 100 times larger than the higher order anisotropies attributed to primordial fluctuations, which is naturally interpreted as the net motion at approximately 370 km s^−1^ of the Solar System barycenter with respect to CMB photons [3]. Analyses of the aberration and correlation of the higher order multipoles suggest that this inferred velocity is not systematically in error (e.g. [4,5]). However, as the surface of last scattering of CMB photons was at z∼1100, while the emission of light from large-scale structure occurred at much later epochs, it is not *a priori* guaranteed that large-scale structure and the CMB should appear to be in the same reference frame—this is the kinematic expectation from the assumption of isotropy that together with homogeneity forms the Cosmological Principle.

The Ellis–Baldwin test is in principle straightforward to carry out once a sufficiently large catalogue of extragalactic objects is in hand. Now that such catalogues are available, there has been an explosion of interest in the test—with over half of citations to the 1984 paper in the last 4 years alone—due to the results being incompatible with the Cosmological Principle at increasingly high significance and adding to a growing list of cosmological tensions captured in several recent and timely reviews [6–9].

The purpose of the present work, as part of this special issue of *Philosophical Transactions A* dedicated to challenges to the standard cosmological model, is to review the Ellis–Baldwin test. In §2, I discuss the physical and mathematical foundations of the test, as well as important considerations such as the effect of local clustering and shot noise. In §3, I summarize the key empirical results, which have revealed a factor of two to three disagreement with the kinematic expectation at a significance level now exceeding 5σ. I comment on the overall state of cosmology in light of these results in §4.

## 2. The Ellis–Baldwin test

### (a) The kinematic dipole

In its original formulation, Ellis & Baldwin derived the expression for the observed anisotropy of distant, discrete objects due to an observer’s peculiar motion at velocity v, which has a corresponding relativistic Doppler factor for angles θ with respect to the direction of motion


(2.1) $$\begin{array}{lcr} & \delta =\gamma (1+\beta \;\cos\;\theta ) & \end{array}$$

where γ is the Lorentz factor and β≡v/c. Given a population of sources with spectral flux densities $S\propto \nu^{-\alpha }$ and source counts per solid angle rising with flux density as $dN(>S)/d\Omega \propto S^{-x}$, the observed source counts above flux density S due to special relativistic Doppler boosting and aberration vary on the sky as


(2.2) $$\begin{array}{lcr} & \frac{dN(>S)}{d\Omega_{\mathrm{obs}}}=\frac{dN(>S)}{d\Omega_{\mathrm{rest}}}\delta^{2+x(1+\alpha )} & \end{array}$$

For velocities much smaller than the speed of light, equation (2.2) reduces to


(2.3) $$\frac{dN(>S)}{d\Omega_{obs}}=\frac{dN(>S)}{d\Omega_{rest}}[1+(2+x(1+\alpha ))\beta \cos\theta ].$$

If these sources are isotropically and homogeneously distributed in comoving volume, then $dN(>S)/d\Omega_{\mathrm{rest}}$ is a monopole and equation (2.3) describes a dipole with relative amplitude


(2.4) D=[2+x(1+α)]β.

For isotropically and homogeneously distributed sources, the *null hypothesis* is that the distribution of sources across the sky for a moving observer above some flux limit will have a dipole amplitude, relative to the monopole, given by equation (2.4), called the Ellis–Baldwin formula.

Note that real, astrophysical sources do not generally have a constant value of x or α across all flux densities, so it may not be clear what value of x or α to use, such as the average value across all S, the value near the flux limit or perhaps a polynomial modification of the Ellis–Baldwin formula. The answer is that the value as close to the flux limit as possible should be used. To see why this is, imagine building a survey one differential unit dS at a time, such as integrating with a detector down to some flux limit. As the survey integrates between S+dS and S, the source counts between S+dS and S will go as N(>S) − N(>S+dS), so the dipole anisotropy from N(>S+dS) is subsumed in N(>S). Thus, once $S=S_{\mathrm{cut}}$, the values of x and α that determine the expected kinematic dipole amplitude are those at $S_{\mathrm{cut}}$, so only the modulation near the flux limit of the survey affects the observed dipole, as intuition suggests. This was rigorously proven by [10], who showed that the Ellis–Baldwin formula holds *exactly*, regardless of any correlation between α(r) and x(r) with comoving distance r (i.e. ‘source evolution’ [11–13]) if x and α are taken at the flux limit of the catalogue. The kinematic dipole described by the Ellis–Baldwin formula is fundamentally a special relativistic effect: *any* isotropic and homogeneous field of discrete sources with spectral indices α and flux distribution proportional to $S^{-x}$ will exhibit an apparent dipole due to observer motion with amplitude given by equation (2.4), independent of any other parameter such as redshift. As Ellis & Baldwin [2] themselves wrote,

*The great power of this test is that the measurements can be made (and the result must hold) for any source counts, whether in a wide or a narrow solid angle, for flat or steep source spectra, etc, irrespective of selection effects or source evolution, as long as the forward and backward measurements are done in the identical manner*.

### (b) The clustering dipole

Homogeneity being a statistical property of the universe complicates the Ellis–Baldwin test, however, because a density field comprised significantly of nearby matter may exhibit a large apparent dipole due to local inhomogeneities. In ΛCDM, extragalactic sources are expected to be clustered following the power spectrum P(k) of matter density perturbations


(2.5) $$\begin{array}{lcr} & C_{l}=b^{2}\frac{2}{\pi }\int_{0}^{\infty }f_{l}(k{)}^{2}P(k)k^{2}dk & \end{array}$$

where b is the linear bias of the sources with respect to matter and $f_{l}(k)$ is the integral over the spherical Bessel function of order l multiplied by a probability function describing the comoving distance to the objects, calculated using their redshift distribution (see the appendix in [14] for details). There is, therefore, an expected ‘clustering dipole’ [15]—not necessarily aligned with the kinematic dipole—that can be of amplitude comparable to or exceeding the kinematic prediction for objects at low redshift. For example, using galaxies selected from the Two Micron Sky Survey, [16] determined the dipole of z≲0.1 galaxies to be D∼0.1, two orders of magnitude larger than the approximately 10^–3^ kinematic dipole (see also [17,18]). Unambiguously detecting the kinematic dipole therefore requires using objects at redshifts exceeding approximately 0.1 where ΛCDM predicts that the kinematic dipole should dominate over the clustering dipole.

While Ellis & Baldwin did not explicitly discuss the clustering properties of radio galaxies, they did note that these sources have redshifts around z∼1. Indeed, predictions for radio sources powered by active galactic nuclei (AGN) based on evolutionary models for radio sources published in the same year [19] indicate that about half should have z>0.8 and only a few percent should have z<0.1 [20]. This has been supported by later work (e.g. [21–25]), but it is worth noting that the spectroscopic redshift distribution of extragalactic radio sources is poorly constrained, limited mainly to a few hundred sources from dedicated follow-up observations (e.g. [26]). This is a consequence of two observational limitations. First, the host galaxies of powerful radio sources at moderate redshift are very faint at other wavelengths. For example, a large elliptical galaxy with an absolute visual magnitude of −22 and a radio spectral luminosity of $10^{25}$ W Hz^−1^ (similar to the nearby galaxy Messier 87) at z=0.5 would be readily detected in a radio survey to a depth of 10 mJy but would have an apparent magnitude at visual wavelengths of approximately 21, too faint for general, wide-area spectroscopic surveys such as the *Sloan Digital Sky Survey* (SDSS; [27])^1^ . Second, even if the host galaxy is near enough for detection by wide area surveys, the majority of the radio power may originate in lobes situated on scales of a hundred kpc from the AGN core, giving arcminute-level angular offsets that make host galaxy identification difficult. Nonetheless, the general consensus is that radio galaxies selected above approximately 10 mJy are predominantly at moderate redshift, so the effect of clustering on kinematic dipole measurements should be minimal (though see [29], [30] for contrasting views).

### (c) The limit set by shot noise

For any finite sample of discrete, uncorrelated sources, the error in N(>S) for any given solid angle element dΩ will be due to shot noise. Ellis & Baldwin [2] give the amplitude of this noise as $D_{\mathrm{SN}}=(2N{)}^{-1/2}$, so that a kinematic dipole of amplitude $D=4.6\times 10^{-3}$, as they estimated for radio sources, would require a catalogue of size $N∼2\times 10^{5}$ to detect at the 3σ level. However, since shot noise is constant with angular power, going as $C_{l,\mathrm{SN}}=4\pi /N$, then for a full, unmasked sky of isotropically and homogeneously distributed sources, the dipole term due to shot noise is


(2.6) $$D_{SN}=\sqrt{\frac{9C_{1,SN}}{4\pi }}=\frac{3}{\sqrt{N}}.$$

Realistically, the sky will be masked to mitigate Galactic contaminants or source confusion. Then, so long as the distribution of sources does not contain significant power from the l>1 multipoles, the shot noise dipole can be estimated as [25,31]


(2.7) $$\begin{array}{lcr} & D_{\mathrm{SN}}∼3\sqrt{\frac{f_{\mathrm{sky}}}{N}} & \end{array}$$

where N is the total number of sources not masked.

Considering that most radio catalogues assembled for the Ellis–Baldwin test have been of a few hundred thousand objects after masking approximately 20−30% of the sky, the kinematic dipole of radio galaxies cannot be detected at greater than the approximately 1σ level, as was shown by [32] and [33] (see also [34]). Therefore, if a dipole is detected at much greater than the 1σ level in current radio catalogues then the dipole *must* be larger than the kinematic prediction, whatever its nature.

## 3. The kinematic dipole disagreement

### (a) Radio galaxies

The publication of the National Radio Astronomy Observatory Very Large Array Sky Survey (NVSS) near the turn of the millennium [20] allowed the first Ellis–Baldwin test of useful statistical power. This was carried out by [15], who reported a dipole close to the expected amplitude corresponding to the approximately 370 km s${,}^{-1}$ kinematic expectation ($D∼4.5\times 10^{-3}$). However, the deepest flux density cut for which the dipole model they employed leaves no unexplained variance in the distribution of radio galaxies shows a dipole with amplitude $D=(1.1\pm 0.3)\times 10^{-2}$, a 2.2σ tension (see their Table 1) that implies a velocity closer to 900 km s${,}^{-1}$.

Subsequent works have found a range of dipole amplitudes in the NVSS (e.g. [16,34,35]), though typically about a factor of three larger than the kinematic expectation. The considerable variation between these results is due to several factors. First, some disagreement exists about what lower flux cut to use. Typically a value of about 15 mJy is used with NVSS data, although the uniformity of a given flux cut depends on the second factor, which is how the sky is masked. Some authors adopt a simple cut in Galactic latitude to mask foreground contaminants (e.g. [35,36]), while others include specific sources or regions to mitigate the clustering contribution of local sources to the dipole, reduce contamination by extended radio sources or remove catalogue systematics (e.g. [15,24,34,37,38]). The effect of removing locally clustered sources appears to be minimal (e.g. [36]), though [30] recently claim otherwise. Another masking strategy is to include a cut on the Galactic synchrotron foreground, as was done in [25,39] and [14].

Finally, how the dipole is estimated varies. A direct multipole expansion can cause power leakage into the higher multipoles for a masked sky, while the ‘linear’ estimator (e.g. [33]) is biased both in amplitude and direction [37,40]. Another approach is to fit a dipole model, employing either a least squares (‘quadratic’) estimator or maximizing a specified likelihood function. While this is relatively unbiased in amplitude and direction, it does force an explicit dipole model that assumes that power in the higher multipoles is negligible compared with the dipole, as expected given the kinematic interpretation of the dipole and linear perturbation theory. A violation of this assumption therefore constitutes a rejection of the null hypothesis by itself, provided that there is not excess power in the higher multipoles due to systematics. For NVSS, this is certainly the case, given both the minimal unexplained variance after fitting a dipole model—at least for flux cuts greater than approximately 20 mJy as found by [15]—and later analyses of the angular power spectrum of NVSS sources [41–43]. Since the NVSS dipole has generally been found to be a factor of three larger than expected, the corresponding statistical significance of the dipole has also been reported to be around ∼3σ, as expected given the few hundred thousand sources used (see §2(c)). More recent analyses have combined NVSS data with radio data from later surveys with southern sky coverage, such as the 843 MHz Sydney University Molonglo Sky Survey (SUMSS; [44]) and the 887.5 MHz Rapid ASKAP Continuum Survey (RACS; [45]), finding a similarly too-large dipole [36,38].

Not every radio survey has given consistent results, however. Using data from the 150 MHz TIFR GMRT Sky Survey (TGSS; [46]), [25] find a dipole three times larger than that of the NVSS, a result confirmed by [37], who consequently argue for a frequency dependence of the anomalous dipole. However, TGSS is known to have systematic flux calibration issues on $10^{\circ }-30^{\circ }$ scales, which manifest both in comparison to TGSS counterparts in the 200 MHz GaLactic and Extragalactic All-sky MWA [47] survey [48,49] and in the presence of anomalously large power in the 2≤l≤30 multipoles, which the NVSS does not exhibit [50]. Indeed, by setting the *average* radio source spectral index to be α=0.75, as is typical at low frequencies, [51] use the NVSS to produce a flux calibration correction map for TGSS, showing that this reduces the TGSS dipole amplitude by a factor of three. Even without correction, however, the TGSS dipole appears to point very closely to that of the CMB, a result that [52] cites to argue that the particularly large dipole in the TGSS has a basis in reality. I agree that it is not immediately obvious why this should be, but emphasize that a dedicated paper on the effect of the TGSS systematics on its apparent dipole has not yet materialized.

The recent 3 GHz VLA Sky Survey (VLASS; [53]) also appears to give results discrepant from previous radio analyses. Combining data from VLASS with RACS to form a nearly full-sky radio catalogue, [54] find a dipole consistent both in direction and amplitude with the kinematic expectation, in contrast with previous results from other radio surveys. However, there is a drop in VLASS source density south of decl. approximately $-15^{\circ }$ [54], and I find that this drop in VLASS source density is not correctable by increasing the flux density cut, unlike the declination-dependent sensitivities of both NVSS and RACS (e.g. [38], figure 1), suggesting that the problem is, effectively, flux calibration error. Indeed, even after masking the sources below $-15^{\circ }$, the VLASS-only dipole points towards the south equatorial pole [54], $80^{\circ }$ from the CMB dipole, in contrast with the other radio surveys that have dipoles pointed near that of the CMB (albeit with anomalously large amplitudes), suggesting that the data above $-15^{\circ }$ are subject to residual systematics. VLASS recently completed its third and final epoch, however, so these issues may be alleviated in the final, coadded data. A thorough assessment of potential systematics in VLASS as they affect power in the dipole and lower multipoles would then be timely.

The presence of certain systematic errors in one catalogue, however, does not logically imply the presence of such errors in another. Indeed, in the over 5000 publications citing the NVSS since its publication 27 years ago I did not find a single report of systematic flux calibration errors, suggesting that any systematic flux calibration errors in this venerable survey are below the sensitivity of an enormous range of scientific applications. The anomalous dipole found with NVSS should therefore be taken seriously. The NVSS result is further reinforced by comparison to the WENSS, SUMSS and RACS catalogues that do not, to my knowledge, have known systematic errors likely to skew measurement of the dipole (as is the case with TGSS and, it would appear, VLASS epoch 1). These catalogues have dipole amplitudes and directions consistent with that of the NVSS for a wide range of flux density cuts and masking strategies (see table 9 in [37], as well as [38]), though I note that only RACS has statistical power comparable to that of NVSS. Combining NVSS and RACS, [38] find that the radio galaxy dipole closely aligns with that of the CMB, though it is nearly three times larger at a significance approaching 5σ.

If matter on large scales shares the same kinematic frame as the CMB and is isotropic and homogeneous—as the Cosmological Principle requires—then there must somehow be a common error in these analyses. Considering the widely varying analysis methods—flux cuts, masking, dipole estimators, etc.—an anomalously large dipole signal almost certainly exists in these data. That leaves either survey systematics, such as those discussed above, or errors of interpretation, such as if contamination from locally clustered sources is more significant than expected (as was recently argued by [29]). The dearth of spectroscopic redshifts for most radio galaxies may appear to allow this, but the main reason for this deficiency, namely that radio galaxies at moderate redshift are too faint for spectroscopic surveys, sets *a priori* limits on how close the galaxies can be. Continuing from the example given in §2(b), a radio galaxy with an absolute visual magnitude of M=−22 mag can have a distance modulus no smaller than ∼40 mag before it likely would have been targeted for the SDSS main galaxy sample [55], corresponding to a lower redshift limit of z∼0.2 (a comoving distance of $570\;h^{-1}$ Mpc), not low enough for the clustering dipole to be significant in the standard cosmology.

Nonetheless, given the profound and foundational consequence that a violation of the Cosmological Principle at the level suggested by studies of radio galaxies would have on FLRW-based cosmologies such as ΛCDM, it is imperative that observational and astrophysical systematics be ruled out as thoroughly as possible. Ideally, what is needed is a large catalogue of a different kind of object, a type of object known to be predominantly at moderate redshift, observed with telescopes and instruments systematically independent of those used to conduct the aforementioned radio surveys, and numerous enough to unambiguously confirm the anomalously large dipole suggested by radio galaxies.

### (b) Quasars

While ‘quasar’, or quasi-stellar object, originally referred to compact, star-like objects with strong radio emission, the term now largely refers to AGN for which the energy liberated from matter in-fall onto the supermassive black hole outshines the entire host galaxy. Indeed, quasars at moderate redshift routinely have bolometric luminosities exceeding $10^{13}$$L_{⊙}$, three orders of magnitude more luminous than a typical, Milky Way-type galaxy. These extreme luminosities make quasars easy to detect even at high redshift, and it has long been established that the peak of quasar activity in the universe occurred around z∼1−2, with the number of luminous quasars per comoving volume declining rapidly to the present (e.g. [56]). These two characteristics of quasars—their extreme luminosities and increasing prevalence with redshift—mean that even without measured redshifts a sample of quasars selected via some other method is, *a priori*, likely to be dominated by moderate redshift objects. However, unlike radio galaxies, quasars are usually readily detected in spectroscopic surveys because of their aforementioned high luminosities, so about one million have recorded redshifts [57]. Quasars that are too faint at visual wavelengths to obtain spectroscopic redshifts are heavily reddened by dust either local to the central engine (the AGN ‘torus’) or on galaxy-wide scales (for a review of AGN obscuration, see [58]). The distribution of observed torus orientations is random, so torus-reddened quasars should share the same redshift distribution as their optically bright counterparts. Galaxy-scale reddening may be an evolutionary effect, raising the possibility of a different redshift distribution; however, recently [59] found no statistically significant difference between the predominantly spectroscopic redshifts of unobscured quasars and the mix of spectroscopic and photometric redshifts of obscured quasars. Consequently, the redshift distribution of quasars is well understood and the clustering dipole is expected to be of order $10^{-4}\;b$ [14]. As radio galaxies and quasars are expected to share the same, comoving frame on large scales, the dipole in the distribution of quasars should therefore provide an independent check that the radio galaxy dipole is not significantly contaminated by clustering. If the radio galaxy and quasar dipoles are consistent then contamination of the radio galaxy measurement by low redshift clustering is unlikely to be a concern.

About 75% of quasar spectroscopic redshifts come from either the SDSS quasar catalogue [60] or spectroscopic pipeline (as compiled in [57]). These objects occupy a footprint covering about 27% of the sky, limiting their utility for the Ellis–Baldwin test, but moreover are selected at visual wavelengths where Galactic reddening uncertainties are likely to dominate the systematic error. Extinction at wavelength λ is a product of the extinction coefficient $A_{\lambda }/A_{V}$, which depends on the source spectral energy distribution for broad passbands, the total-to-selective extinction ratio $R_{V}=A_{V}/E_{B-V}$, a function of dust composition that varies with line of sight through the Galaxy, and the colour excess $E_{B-V}$, which is typically estimated from Galactic dust temperature. An Ellis–Baldwin test using an optically selected quasar catalogue will be strongly affected by this systematic uncertainty, which may limit the utility of such a catalogue for the test.

Fortunately, there is another, more powerful means of selecting quasars. The *Wide-field Infrared Survey Explorer* (WISE; [61]), a NASA Medium-Class Explorer mission launched in 2009, performed a full sky survey at 3.4 μm (W1), 4.6 μm (W2), 12 μm (W3) and 22 μm (W4), before carrying on as the post-cryogen Near-Earth Object WISE (NEOWISE) mission [62,63], which continued to survey the sky in W1 and W2 until the end of July 2024. A unique property of mid-infrared data taken in these bands is that extraordinarily pure samples of quasars—especially with respect to contamination by stars—can be selected on the basis of photometry alone [64]. This is because quasars have nearly power–law spectral energy distributions in the mid-infrared with spectral indices near α∼1 ($F_{\nu }\propto \nu^{-\alpha }$) due to emission dominated by dust heated to the sublimation limit (approximately 1500 K) by the direct quasar continuum. This produces very red W1–W2 colour, typically around W1–W2 ∼1, across a wide range in redshift. In contrast, stellar emission follows the Rayleigh-Jeans tail with α=−2, giving W1–W2 ∼0 because WISE magnitudes are reported in the Vega system. Dust heated in star forming regions is generally cool, usually a few tens of K, and star-forming galaxies typically have red W2–W3 colour but W1–W2 colour remaining close to that of stars. Thus, a simple cut on W1–W2 can effectively remove both Galactic stars and low redshift galaxies, leaving quasars.

This was the method employed by [14], who performed the first Ellis–Baldwin test using quasars by selecting sources from the CatWISE2020 catalogue [65], the deepest catalogue of WISE photometry yet released, applying the W1–W2 cut from [66], which selects AGN-dominated objects. Using a least squares fit, they found that the quasar dipole has an amplitude over twice as large as the kinematic expectation: $D=1.55\times 10^{-2}$, pointed in a direction $28^{\circ }$ away from the CMB dipole, at a significance level of 4.9σ based on simulated skies. This was not only the most formally significant disagreement with the kinematic expectation reported in the literature at the time: it was also (much more importantly) the first *completely systematically independent* confirmation of the anomalous dipole indicated by studies using radio galaxies because WISE shares no instrumental or calibration systematics with ground-based radio surveys; it was conducted at a completely different wavelength dominated by a different physical mechanism (thermal emission instead of synchrotron), and quasars are almost entirely a different set of objects from radio galaxies, generally residing in bluer hosts with almost no overlap with radio-selected AGN (e.g. [67], see also [68]). The near-total population independence of radio galaxies and mid-infrared quasars was formalized in the context of the Ellis–Baldwin test by [51], who removed the residual 1.4% of radio galaxies from the quasar sample to create two completely independent samples with which the joint significance of the anomalous dipole could be assessed, as well as its amplitude and direction under varying assumptions. Using a generalized definition of the *p*-value that is maximally conservative, they rejected the kinematic interpretation of the dipole at 5.1σ, again the most significant result reported in the literature at the time. [69] confirmed these findings, independently reporting a 5.7σ rejection using a Bayesian method applied to the [14] quasar sample, while the 5.1σ result reported by [51] remains the most significant rejection of the null hypothesis based on a frequentist method.

Finally, the new Quaia catalogue [70], created by combining optical spectrophotometry of Gaia DR3 quasar candidates [71] with mid-infrared photometry from the unWISE catalogue [72], may allow for constraints to be placed on the kinematic dipole at visual wavelengths, which would provide a quasi-independent measure of the quasar dipole (using a cut on visual instead of infrared flux) and potentially also allow for redshift tomography, given the spectrophotometric redshifts available in Quaia. A first attempt at measuring the Quaia dipole was published by [73], who recover, for a very strict $|b|<40^{\circ }$ cut on the shallower version of the catalogue, a dipole pointed near the direction of the CMB with amplitude $D=1.1_{-0.5}^{+0.6}\times 10^{-2}$, consistent with both the radio galaxy and quasar dipoles found in previous works, suggesting that Quaia is sensitive to the dipole at some level. [73] initially reported that the kinematic expectation is nonetheless the favoured model, but this was due to incorrectly estimating the spectral indices of the Quaia sample, which led to a significant overestimate of the kinematic expectation of D=0.008. This was recently addressed in an erratum [74], wherein the corrected kinematic expectation is D=0.0048. The measured dipole is thus a factor of two larger than the kinematic expectation, in agreement with the WISE-based quasar results. Moreover, [74] find that the statistical preference for the kinematic expectation is strongly reduced, with the $D=1.1\times 10^{-2}$ dipole being favoured over the kinematic expectation under a different choice of priors. While the Quaia sample more certainly favours the dipole being aligned close to that of the CMB, whether its amplitude is consistent with the kinematic expectation is much less certain. This is unsurprising given the large uncertainty of the Quaia dipole amplitude, a consequence of the large systematic uncertainties introduced at visual wavelengths from Galactic reddening.

## 4. Final remarks

The last two decades have seen the gradual accumulation of evidence that the dipole anisotropy apparent in the distribution of moderate redshift matter is incompatible with the kinematic expectation, given the Cosmological Principle and the inference that the Solar System is moving at approximately 370 km s^−1^ with respect to the CMB. Studies using radio galaxies, catalogued in surveys such as the NVSS, RACS, SUMSS and the TGSS have consistently found a dipole at least twice as large as expected, at the approximately 2−3σ level for single-survey studies, reaching as high as 4.8σ in a recent joint analysis of radio data [38]. Studies using quasars have robustly confirmed the dipole anomaly at a level exceeding 5σ. Given these results, and considering the near-total systematic independence of radio galaxy and quasar catalogues, including with respect to shared sources, it is increasingly difficult to imagine how the kinematic dipole problem could be a result of systematics or methodological errors.

This highlights a somewhat peculiar phenomenon in the cosmological literature. The dipole tension and the ‘Hubble tension’ formally reached the 5σ level at almost exactly the same time, with both results being reported in *The Astrophysical Journal Letters* within the span of a few months ([75] and [51], respectively). Both tensions point to foundational problems with ΛCDM, yet the Hubble tension receives, by far, most of the research effort, a curious state of affairs recently remarked upon in a review by [7]. A potential reason for this discrepancy is simply the large number of methods that have been developed for measuring $H_{0}$ which do not generally agree (for a recent review, see [76]), motivating a wide range of theoretical proposals to resolve the tension (for reviews, see [77,78]). In contrast, there are only a handful of datasets that have proven useful for measuring the dipole of moderate redshift matter, slowing progress on the empirical front until data from new surveys such as Euclid, SPHEREx or the Square Kilometre Array become available. In the meantime, tighter independent constraints on the kinematic dipole may be made with data from the Dark Energy Spectroscopic Survey [79], which across its approximately 14 000 square degree footprint records the spectroscopic redshifts of millions of galaxies out to moderate redshift. If the Solar System barycenter velocity with respect to moderate-z matter is tightly constrained to be close to the 370 km s^−1^ value indicated by the z∼1100 CMB, then radio galaxies and quasars may exhibit an intrinsic dipole anisotropy potentially bearing no directional relation to that of the CMB, as hinted at by [51].

Practical or observational considerations aside, an anomalous dipole of moderate redshift matter is fundamentally incompatible with the FLRW metric, so must be addressed. If indeed the assumption of statistical homogeneity or isotropy underpinning ΛCDM—and all FLRW cosmologies— is violated, then the consequences for cosmology may be profound. The exact solution to the Einstein equations afforded by the Cosmological Principle allows for the universe to be uniquely specified by just a few parameters (in the case of the standard ΛCDM, six). In the limit of sparse data and computational constraints, this has enabled tight estimates of these model parameters and their posteriors, ushering in the age of ‘precision cosmology’ with the advent of the Cosmic Background Explorer, the Wilkinson Microwave Anisotropy Probe, and Planck. But precision is not the same as accuracy and even extremely precise statistical constraints on the cosmological parameters cannot take into account errors arising from model choice.

Nonetheless, ΛCDM has for several decades been able to comfortably accommodate the majority of cosmological observables. It is, therefore, understandable when apparent tensions with ΛCDM are treated with scepticism. Indeed, the undoubted success of ΛCDM in explaining the CMB—anomalies notwithstanding (e.g. [3])—as well as predicting the value of the current Hubble parameter to within 10% (as pointed out by [7]) means that one naturally assumes a reasonable prior against tensions that are formally statistically significant. Certainly, any extension to or replacement for ΛCDM must reproduce its successes in the domain of previous data, as is a requirement for any proposed scientific model.

## Data Availability

This article has no additional data.

## Footnotes

^1^
Using h = 0.7 and calculating the K correction from Table 2 in [28]
